# Design and evaluation of a solar powered smart irrigation system for sustainable urban agriculture

**DOI:** 10.1038/s41598-025-94251-3

**Published:** 2025-04-06

**Authors:** Mahmoud A. Abdelhamid, Tarek Kh. Abdelkader, Hassan A. A. Sayed, Zhao Zhang, Xiaohui Zhao, Mohamed F. Atia

**Affiliations:** 1 Department of Agricultural Engineering, Faculty of Agriculture, Ain Shams University, Cairo, 11241 Egypt; 2 Solar Energy Research Institute, Yunnan Normal University, Kunming, 650500 China; 3 Department of Agricultural Engineering, Faculty of Agriculture, Fayoum University, Fayoum, 63514 Egypt; 4 School of Energy and Environment Science, Yunnan Provincial Rural Energy Engineering Key Laboratory, Yunnan Normal University, No. 768 Juxian Road, Chenggong University Town, Kunming, 650500 P. R. China; 5 Department of Agricultural Power and Machinery Engineering, Faculty of Agricultural Engineering, Al-Azhar University, Cairo, 11751 Egypt; 6 Key Laboratory of Agricultural Information Acquisition Technology, Ministry of Agriculture and Rural Affairs, China Agricultural University, Beijing, 100083 China; 7 International Office, China Agricultural University, Beijing, 100083 China

**Keywords:** Food security, Solar energy, Intelligent sensors, Irrigation system, Smart agriculture, Rooftop, Agroecology, Electrical and electronic engineering

## Abstract

Urban areas face significant challenges, including a lack of green spaces, scarce water resources, environmental pollution, and elevated heat emissions, particularly in developing countries experiencing rapid population growth. Therefore, the study aims to advance sustainable urban agriculture by designing and evaluating a solar-powered smart rooftop irrigation system for peppermint cultivation. The system incorporates two drip irrigation setups—conventional and smart irrigation—powered by photovoltaic (PV) panels. The smart system integrates real-time monitoring of critical variables, including (1) soil moisture, (2) relative humidity, (3) PV panel temperature, and (4) PV panel current and voltage. Key performance metrics such as water and energy consumption, water use efficiency, energy productivity, and carbon dioxide emissions were evaluated for both systems. In addition, the economic analysis of the smart system was determined. Results revealed that the smart system reduced water and energy consumption by 28.1% compared to conventional irrigation. Additionally, the smart system achieved a notable reduction in carbon footprint, with CO_2_ emissions of 0.181 kg CO₂/m^2^/year compared to 0.252 kg CO₂/m^2^/year for the conventional system. The system’s economic analysis demonstrated a payback period of 5.6 years, highlighting its financial viability. This study underscores the transformative potential of solar-powered smart irrigation systems in enhancing food security, conserving water, reducing energy consumption, and mitigating carbon emissions in urban agriculture.

## Introduction

The current population growth trends result in a rise in the need for energy, water, and food^1^. This need becomes problematic, especially for cities, which are home to half of the world’s population and are predicted to increase to over 70% by 2050^2^. Agricultural sustainability is a very critical issue^3–5^. Urban agriculture (UA), as a multifunctional system^6^, significantly contributes to urban development challenges^7^. UA fundamentally contributes to household food provision^8^ and encompasses additional food-related welfare dimensions, including enhancing food security and fortifying the food system’s resilience^9^. Moreover, growing research illustrates urban agriculture’s social, economic, and environmental advantages beyond just food production^6,10,11^. These supplementary values include enhancing community ties^12^, offering educational prospects, augmenting soil water infiltration, alleviating the urban heat island phenomenon^13^, and optimizing the use of urban organic waste, among others^14^. Urbanization, climate change, equitable economic models, and health challenges propel UA from the periphery to the forefront of discourse^15^. UA offers opportunities to improve urban metabolism and promote resource circularity. UA predominantly exerts beneficial effects on numerous Sustainable Development Goals: 1, 2, and 4 (quality education); 5 (gender equality); 8 (decent work and economic growth); 9 (industry, innovation, and infrastructure); 10 (reduced inequalities); 11, 12, 13, 15, and 16 (peace, justice, and strong institutions); and 17 (partnerships for the goals). They embody the three pillars of sustainability: society, economics, and environment, emphasizing urban agriculture’s potential for comprehensive sustainable development beyond only improving food security^16^. Socio-economic development has expedited urban expansion, resulting in the encroachment of urban areas into agricultural land and significantly compromising ecosystem stability. Scientifically improving the configuration of UA areas can ensure food security, foster urban development, and preserve ecological stability^17^. In other words, since additional resources must be imported, cities cannot increase their food sovereignty at the expense of worsening environmental effects. UA is frequently marketed to mitigate these effects while offering numerous advantages for health and well-being^18–20^. At the municipal level, this sharing of resources and places is a useful application of sustainable urban development for the production of food and another multipurpose service^21,22^. The city could promote food self-sufficiency by growing and eating veggies locally^23^. Countries with densely populated cities seek to encourage citizens to use the rooftop farming system. To investigate how green roof systems can be used to lessen the consequences of climate change, a variety of sustainable approaches to energy- and water-saving practices have been created and tested^24^.

Agricultural water consumption, accounting for 70–80% of worldwide water usage, has significant challenges due to climate change, less rainfall, and a growing population^25^. The water crisis is one of the main issues facing the agricultural sector in dry and semi-arid regions^26,27^. The demand for sustainable, water-efficient, and data-driven irrigation systems is critical to guarantee the region’s food security and economic advancement. To improve water-use efficiency and support Sustainable Development Goals (SDGs), especially Goal 6 and Target 6.4, innovative irrigation techniques are necessary^28^. Manual watering of crops is both time-consuming and inefficient, whereas irrigation reliant on fixed timers fails to consider variations in weather or soil conditions that influence moisture levels^29,30^. Intelligent irrigation systems consider meteorological and soil variables to enhance irrigation efficiency, yielding substantial water savings of up to 30 percent^31–34^. Smart irrigation scheduling is a promising method for enhancing the efficiency and sustainability of water for agriculture utilization, particularly in arid and semi-arid regions^35^. The regulation of irrigation is essential for executing smart irrigation systems^33^. Drip irrigation systems, which provide up to 95% irrigation efficiency, are one example of a technology and management technique that maximizes water consumption^36^. Global population growth necessitates increased food production. However, the changing climate results in less available water, which causes energy and water shortages in all sectors, mostly agriculture^37^. An increasingly popular irrigation technique worldwide is smart irrigation, which is state-of-the-art and eco-friendly. Smart irrigation systems using cutting-edge technology and data analytics can increase agricultural yields, improve plant quality, and help conserve water. Despite many barriers to its widespread usage, smart irrigation is a workable solution for the issues brought on by climate change and water constraints^38^. Farmers may save labor costs and enjoy convenience with smart irrigation control systems. Farmers can reduce the requirement for manual intervention by remotely monitoring and controlling their irrigation systems through automation of the irrigation process^31^. Farmers may concentrate on other important duties thanks to this automation, which also saves time and effort and increases operational efficiency^31^. Numerous research works have documented the use of soil moisture sensing for irrigation monitoring^39–43^.

Global energy demand consistently increases due to technological advancement, contemporary lifestyles, and population expansion^44^. Solar energy is the most potential alternative energy source due to its availability, sustainability, and environmental benignity^45–47^. Solar irrigation systems should become increasingly feasible and efficient with technological advancements^48^. Solar power aims to significantly enhance global energy supply in light of the limited availability of fossil fuels and growing awareness of environmental degradation^49,50^. Recently, there has been a lot of interest in solar-powered water pumping devices. There is great potential for developing a solar-powered smart irrigation control system kit, especially considering the increasing need for sustainable agricultural techniques. This kit can run independently by using solar energy, which lessens reliance on traditional energy sources and lowers operating expenses for farmers. Moreover, integrating intelligent control algorithms ensures accurate water delivery based on real-time soil moisture data, maximizing water utilization and improving irrigation efficiency^51^. Several studies have been conducted to design and evaluate renewable-driven polygeneration systems capable of producing multiple energy vectors, including electricity, space heating and cooling, hydrogen, oxygen, pure water, domestic hot water, and electric vehicle charging. The effectiveness of these systems relies on the optimal integration of energy conversion devices with a multi-tiered backup system^52,53^. Numerous scholarly articles have examined the enhancement of solar-powered smart irrigation system performance^51,54–59^. Pressures on the effective provision and utilization of water, energy, and food (WEF) have arisen as a challengingly interwoven WEF Nexus due to the fast-increasing population and increased urbanization. This is especially true when the interests of these resources are competing with one another^60^. Wright et al.^24^ addressed the WEF nexus by producing food in cities, improving insulating qualities, and retaining more water.

UA faces significant challenges, particularly its reliance on manual irrigation systems often powered by non-renewable energy sources. This research addresses these challenges by designing and implementing a cost-effective, small-scale automated irrigation system powered by solar energy. Unlike many studies that rely solely on simulation, this work demonstrates the development and evaluation of a rooftop solar-powered irrigation control system that maximizes the efficiency of photovoltaic (PV) energy utilization. The proposed system integrates real-time soil moisture feedback to enable precise irrigation scheduling, significantly reducing both water and energy consumption. By addressing critical gaps in affordable, field-ready smart irrigation technologies, this research strengthens the vital WEF nexus and underscores the transformative potential of renewable energy in sustainable urban agriculture. The study also highlights the broader agricultural impacts of adopting such innovative systems, offering scalable solutions for future food security and resource conservation.

## Materials and methods

### Site information and crop selection

**Fig. 1:**
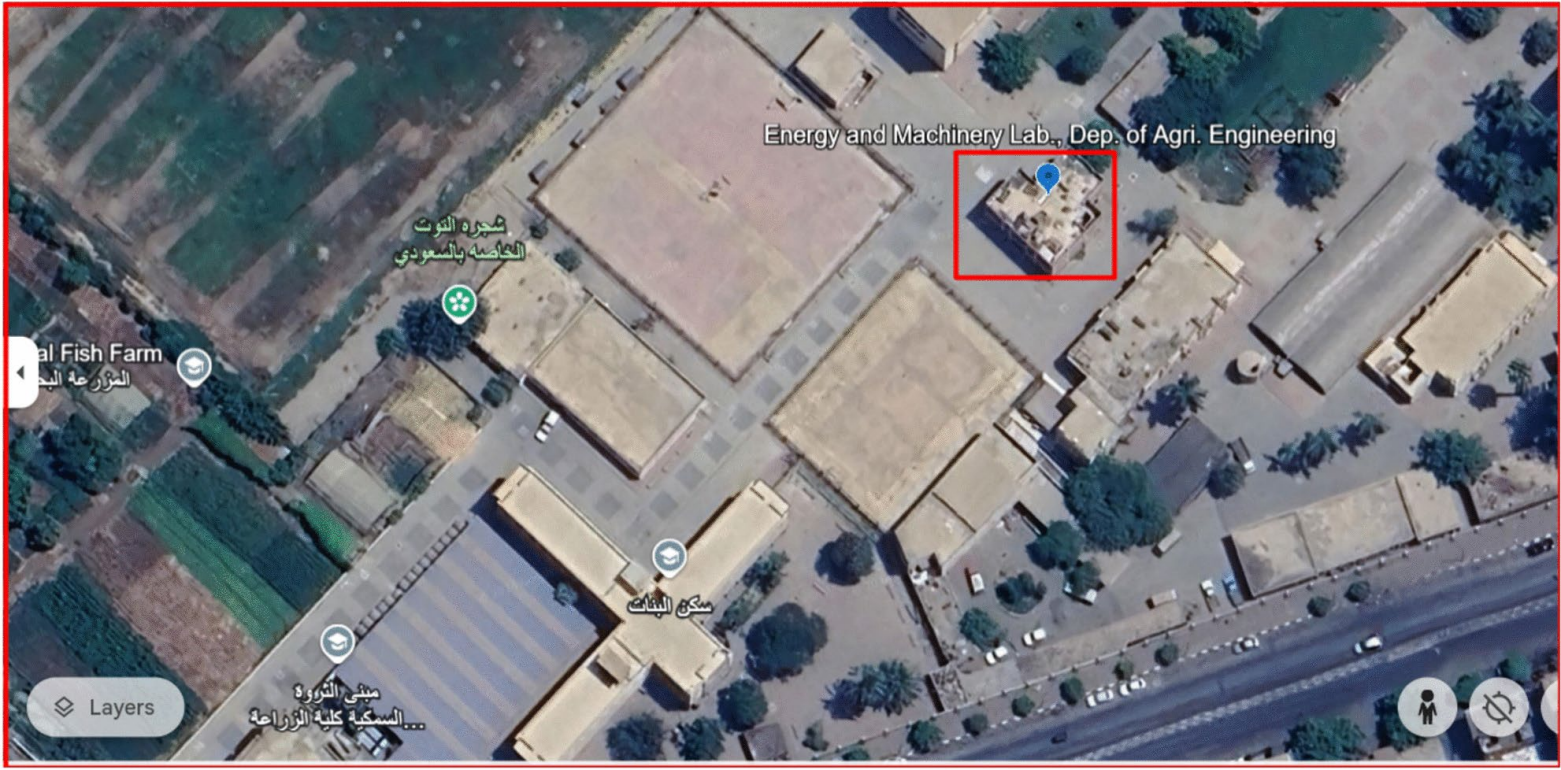
Site location—Energy and Machinery Laboratory, Department of Agricultural Engineering, Faculty of Agriculture, Ain Shams University, Egypt. Source: https://earth.google.com.

The site chosen for the study was the Faculty of Agriculture, Ain Shams University, Egypt (Fig. 1). The Faculty of Agriculture is one of the largest colleges in Egypt. It contains many educational, administrative, and service buildings and student housing. These buildings have a huge amount of roof space available. The experiment was carried out on the roof of the Energy and Machinery Laboratory, Department of Agricultural Engineering, Faculty of Agriculture, Ain Shams University (Latitude 30° 06′ N, Longitude 31^o^ 14′ E).

**Fig. 2:**
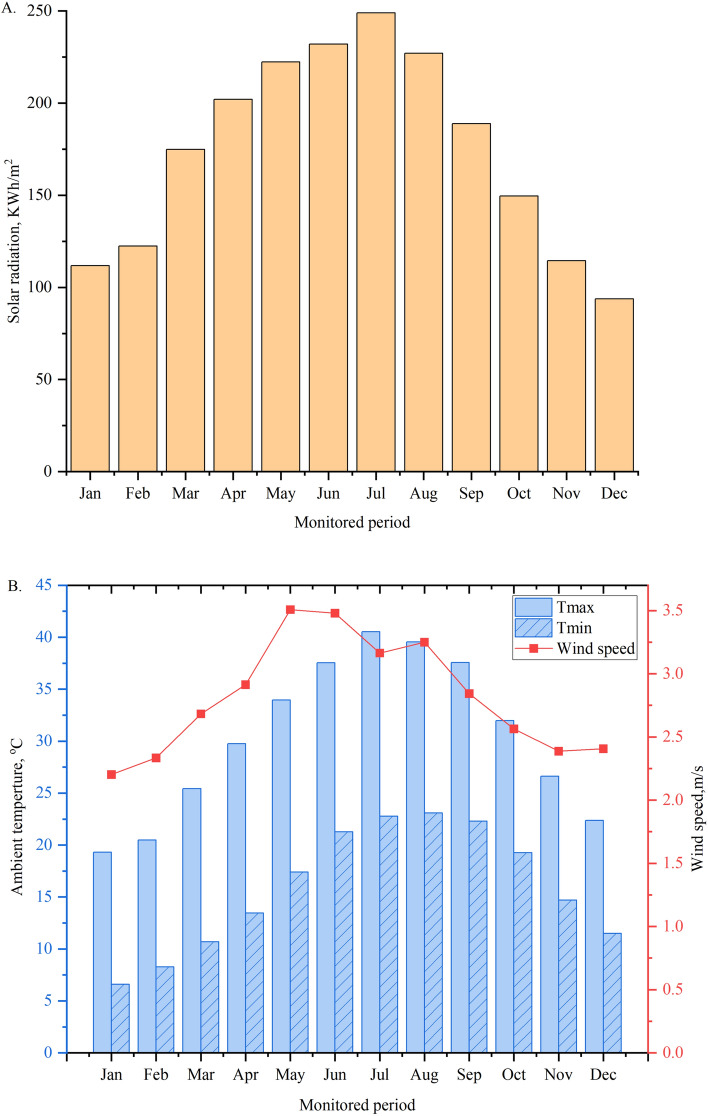
(**A**) Monthly incident solar radiation of the region study and (**B**) The ambient parameters during 2023.

Daily meteorological data of the study area during 2023, including minimum and maximum temperatures (Tmin and Tmax, °C), solar radiation (kWh/m^2^), and wind speed (m/s) were obtained from https://power.larc.nasa.gov/data-access-viewer/. Figure 2A illustrates the region’s average monthly solar radiation for 2023. The data highlights that the maximum solar energy, 248.9 kWh/m^2^, was recorded in July, while the minimum value, 93.7 kWh/m^2^, occurred in December. Figure 2B shows the region’s average monthly maximum and minimum ambient temperatures throughout the year. The highest temperature, 40.5 °C, was observed in July, whereas the lowest, 19.3 °C, was recorded in January. Additionally, the average ambient temperature peaks in July, aligning with the maximum solar radiation levels.

Mint was cultivated on rooftops for its aesthetic appeal and ability to provide dense vegetative cover, protecting buildings from thermal and environmental stresses. Healthy mint seedlings from the Faculty of Agriculture, Ain Shams University, Egypt, were planted in January 2023 in sandy clay soil, chosen for its favorable drainage and water retention properties. The soil was placed in wooden boxes (1 × 2 m) to ensure adequate space for growth. The cultivation followed the faculty’s expert planting and fertilization guidelines, ensuring optimal plant health. A drip irrigation system was employed for efficient water delivery, maintaining consistent soil moisture, and minimizing water loss. The experiment took a year from the beginning of planting. This approach highlights the integration of sustainable UA with functional and environmental benefits.

### System description

**Fig. 3:**
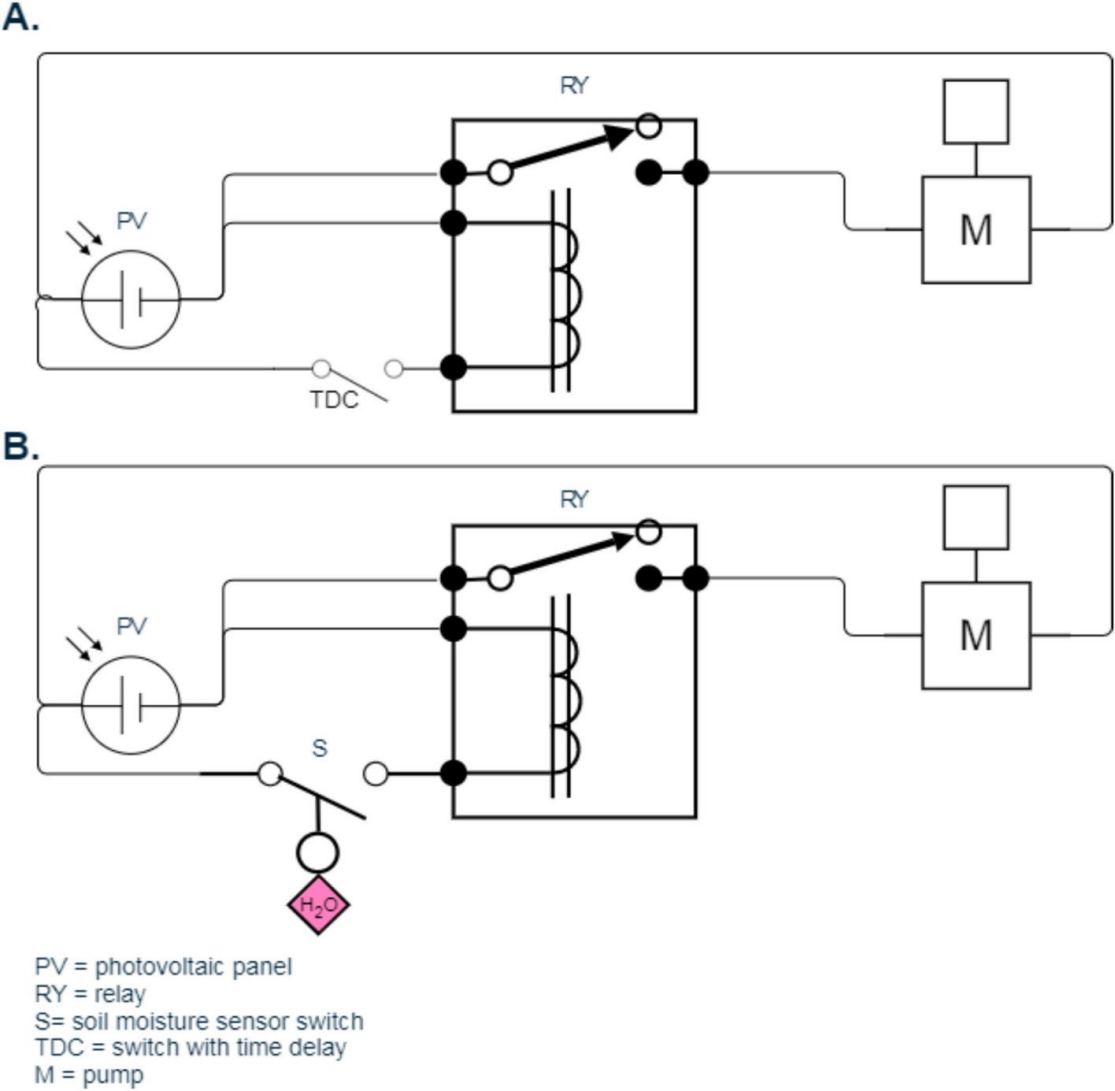
Schematic of the circuit of the conventional system (**A**) and the smart system (**B**).

**Fig. 4:**
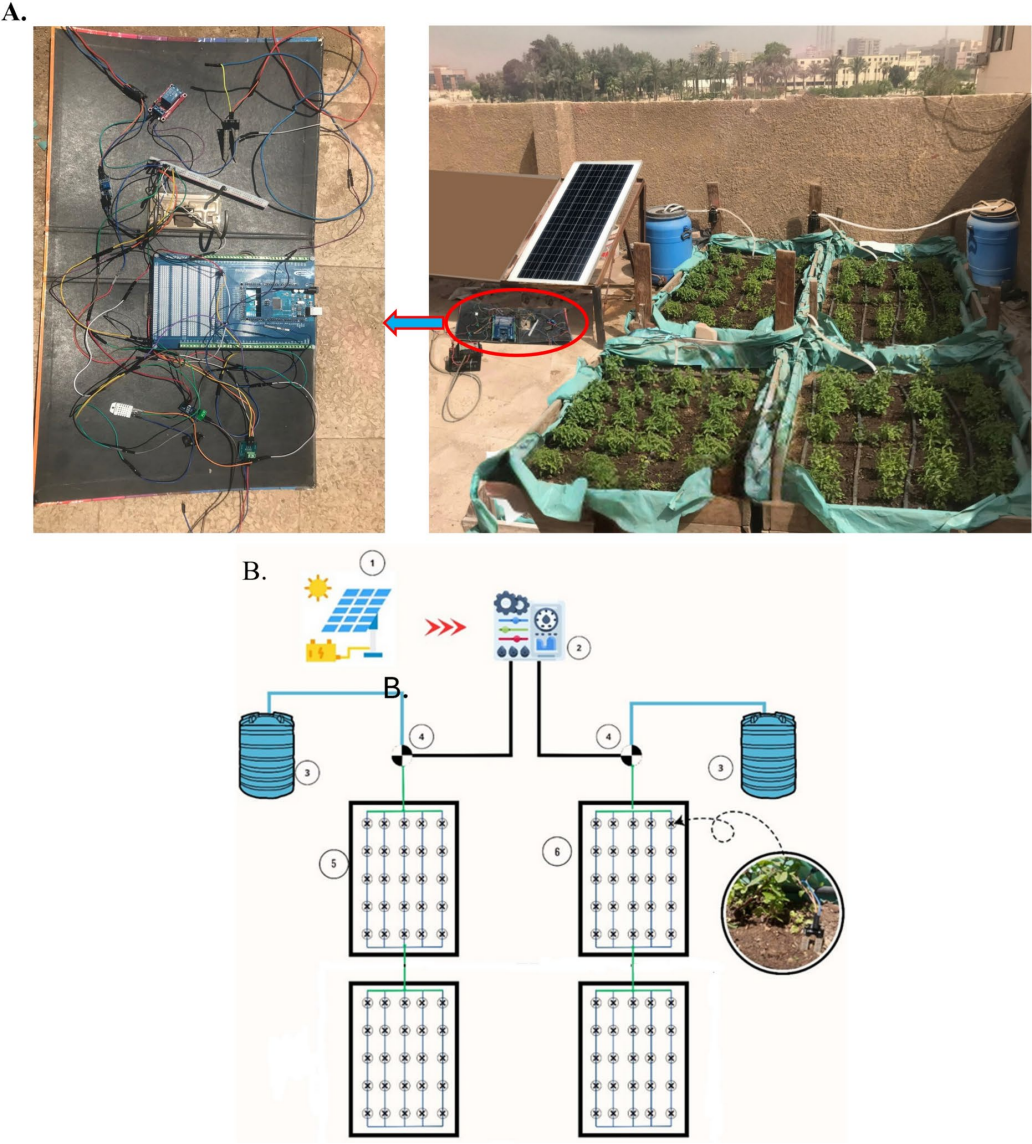
Photographic (**A**) and schematic view (**B**) of the experiment (1—Solar PV, 2—control units, 3—tanks, 4—pumps, 5—conventional irrigation, 6—smart system). The digital drawings in this figure were created by M. A. Abdelhamid using Microsoft PowerPoint (Version 2016, https://www.microsoft.com).

To maximize surface utilization for food production and climate change adaptation, two systems were implemented for cultivating peppermint, both relying on drip irrigation. The first system employed periodic manual irrigation (conventional irrigation) as shown in Fig. 3A, while the second utilized a smart irrigation approach, incorporating a soil moisture sensor for automated control as shown in Fig. 3B. Both systems were powered by solar energy, operating DC irrigation pumps. Figure 4 shows the photographic and schematic of the experimental setup. The system consists of (1) PV solar modules for renewable energy supply to power the entire system, (2) Control units for managing irrigation schedules and sensor inputs, (3) Water tanks, (4) DC water pumps, and (5) Four wooden planting boxes with well-prepared soil for peppermint cultivation.

**Fig. 5:**
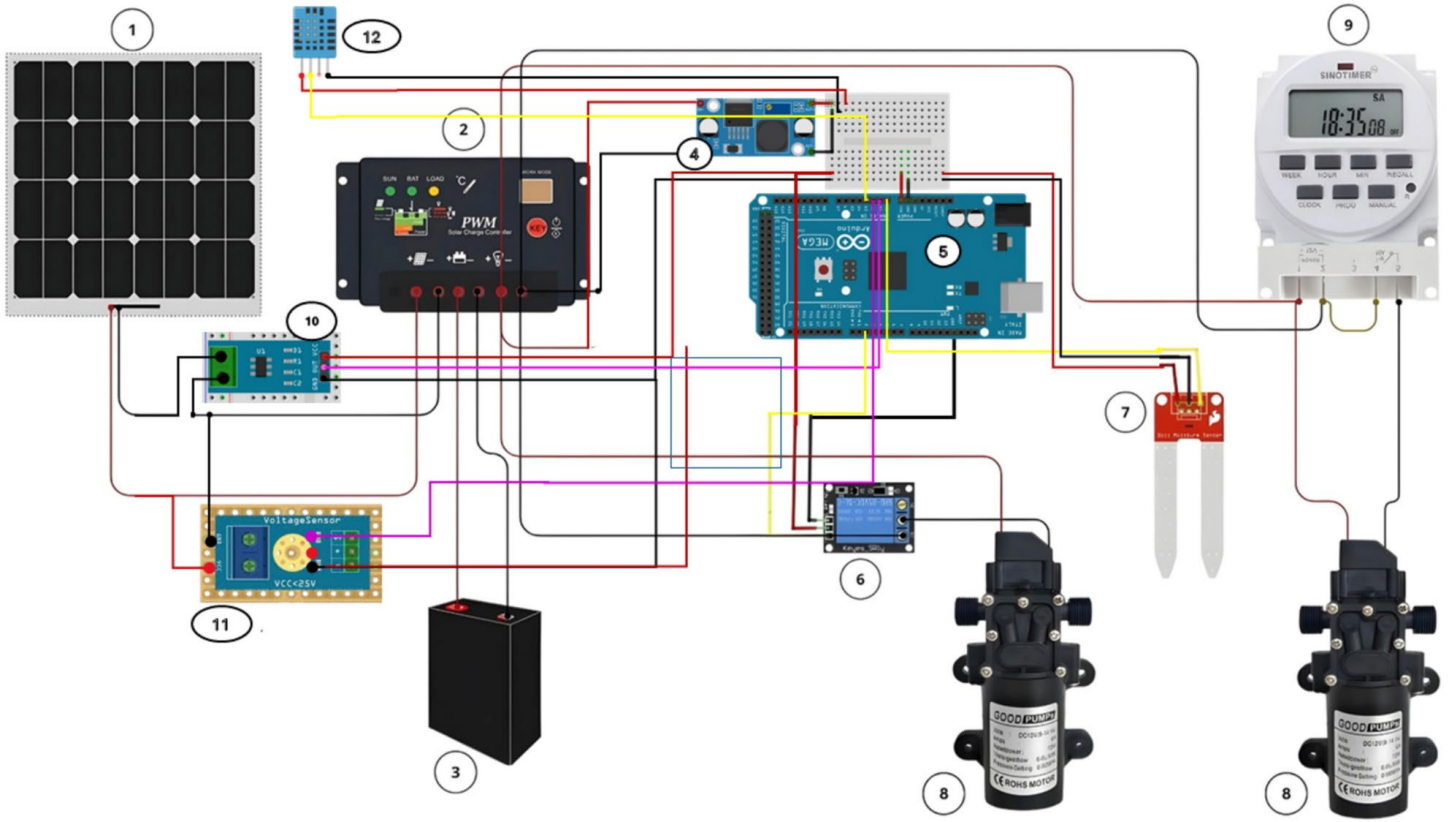
The schematic design of the Arduino-controlled system: 1—solar PV, 2—solar charge controller, 3—12 V battery, 4—DC–DC Buck converter step down module LM2596, 5—Arduino mega 2560 Rev3, 6—relay, 7—soil moisture sensor, 8—DC pump, 9—DC 12V Digital Timer, 10—current sensor, 11—voltage sensor, 12—DHT11 temperature and humidity sensor. The digital drawings in this figure were created by M. A. Abdelhamid using Microsoft PowerPoint (Version 2016, https://www.microsoft.com).

**Fig. 6:**
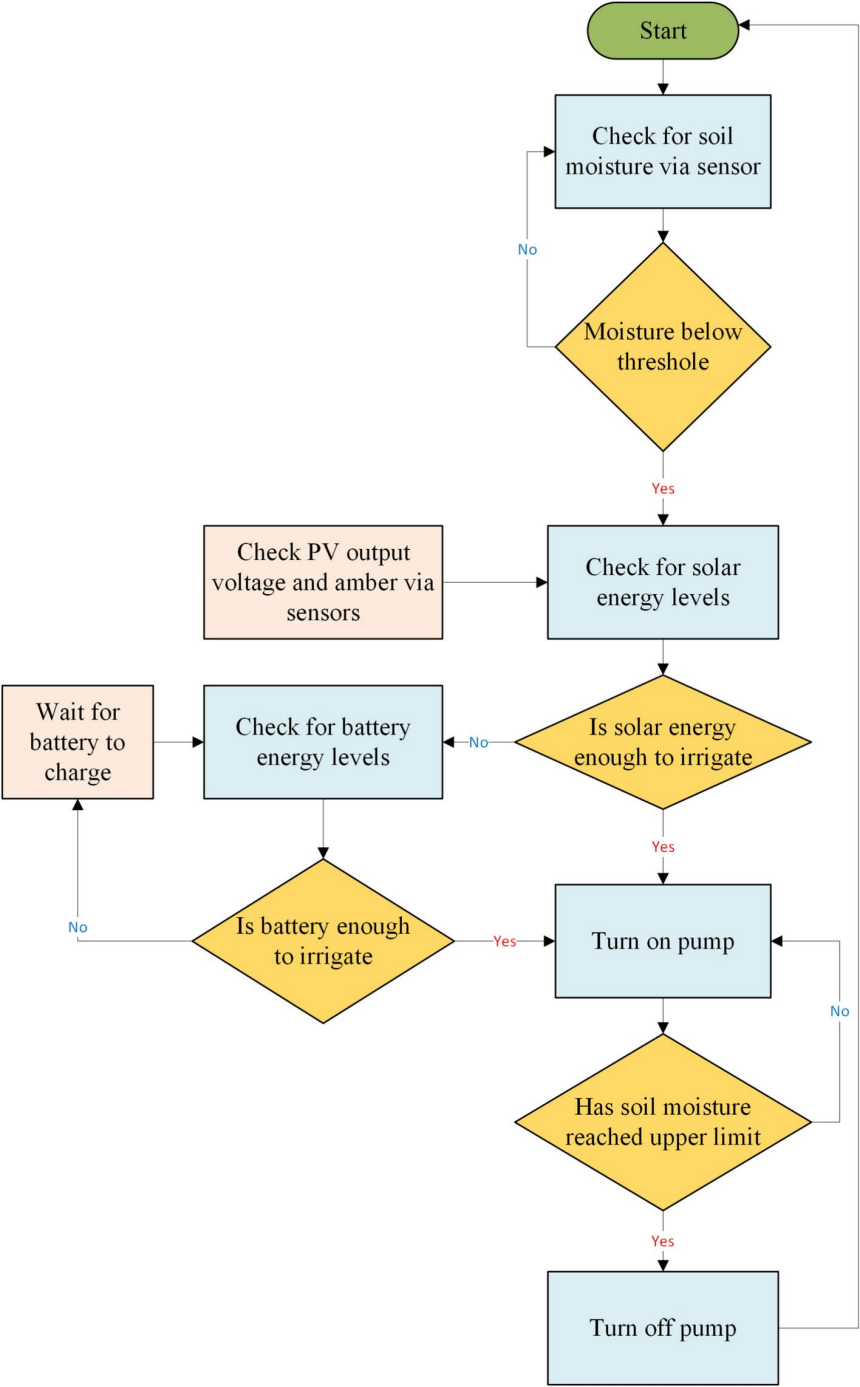
Working flowchart of the smart irrigation system.

**Fig. 7:**
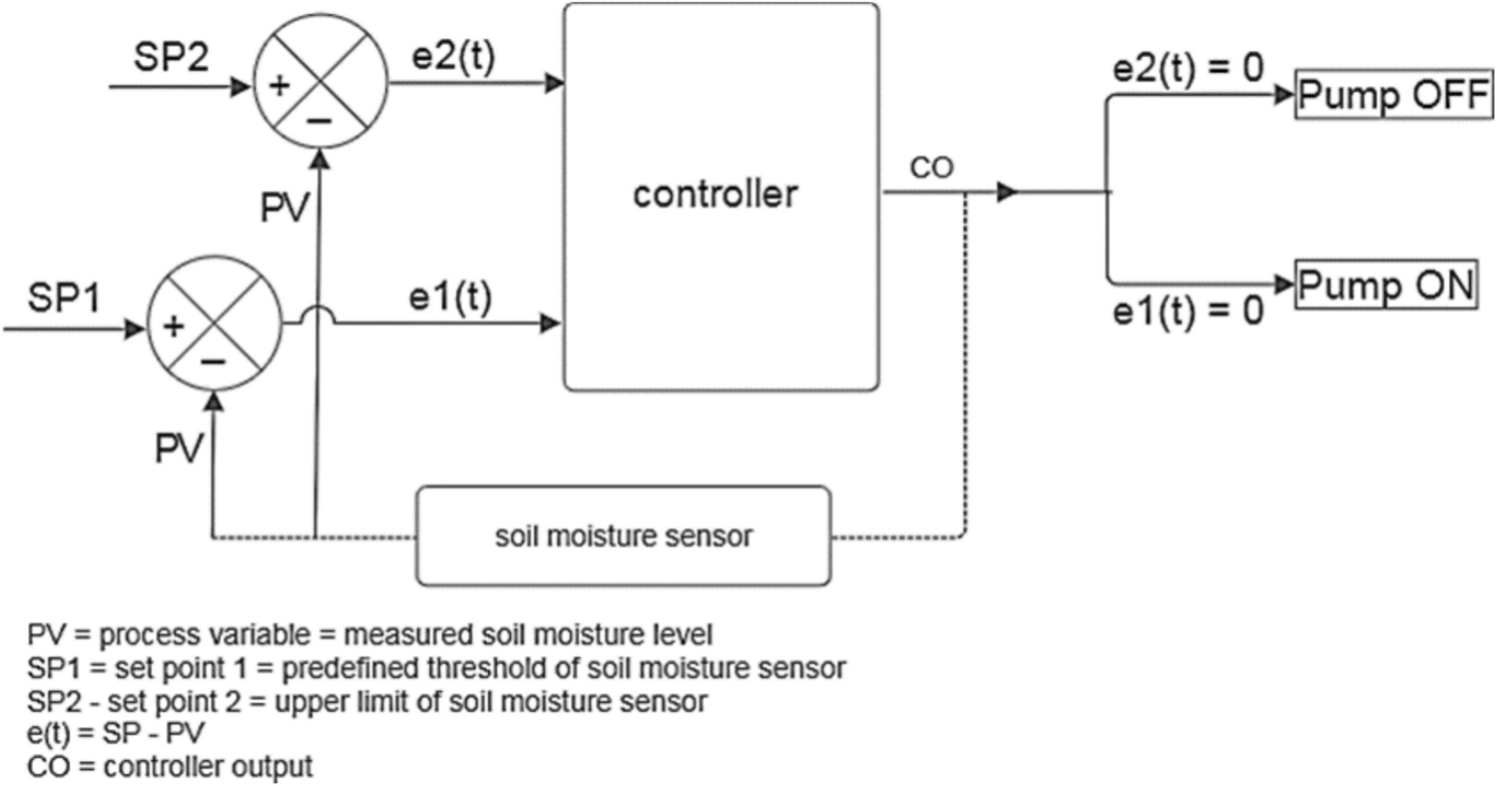
Block diagram of control of pump switching.

In the conventional irrigation system, the pump operates on a predefined schedule, influenced by weather conditions and the crop’s growth stage. In contrast, the smart irrigation system utilizes a soil moisture sensor to trigger the pump, receiving real-time signals from a control unit. A 100-W polycrystalline PV panel is used to operate the pumps, while an Arduino-embedded controller manages the monitoring and operation of the water pumps. Several parameters are monitored, including ambient temperature, PV surface temperature, soil moisture content, PV current output, and PV voltage output. The schematic design of the Arduino-controlled system is shown in Fig. 5. The relay control manages the DC water pumps, with the Arduino controller initiating on/off commands for precise irrigation management. Figure 6 shows the system’s working flowchart. The flowchart illustrates the operation of a solar-powered smart irrigation system designed to maximize water and energy efficiency. The process begins with a soil moisture sensor monitoring the moisture level in the soil. If the moisture falls below a predefined threshold, the system evaluates the availability of solar energy. If solar energy is insufficient, it checks the battery’s energy levels, pausing operation to allow the battery to recharge if necessary. Once adequate energy is available, the system activates the water pump to irrigate. During operation, the soil moisture is continuously monitored, and the pump is deactivated as soon as the moisture reaches the upper limit, the process control is shown in Fig. 7. This automated process seamlessly integrates renewable energy with real-time feedback to ensure precise irrigation, reducing resource wastage and supporting sustainable agriculture.

### Controller system description

An automated system for UA offers a transformative solution to improving the efficiency of both irrigation and energy management. By leveraging advanced technologies, the system can optimize resource utilization, reduce wastage, and enhance agricultural productivity. The following components and functionalities are proposed to achieve these goals:

#### Arduino Mega and software

The embedded board utilized in this study is the Arduino Mega, a microcontroller board based on the ATmega2560. It may be programmed using the Arduino IDE software. It features four hardware serial ports (UARTs), sixteen analog inputs, and fifty-four digital input/output pins, fifteen of which can be utilized as PWM outputs. The Arduino code was written, and all sensors were monitored and recorded in an Excel sheet.

#### Soil moisture sensor

A soil moisture sensor is a specialized device designed to measure the moisture level in the soil, playing a crucial role in gardening and agricultural monitoring. It detects a material’s electrical resistance when it comes into touch with it. Soil moisture sensors detect the electrical resistance between two probes inserted into the soil. Wet soil gives a lower resistance measurement than dry soil because it is more electrically conductive. The resistance reading can be used to estimate the soil’s moisture content.

#### Relay

An electronic switch that uses a low voltage signal to operate circuits with high voltage and high current is called a 5V relay module. The 5V relay module gets its name from the voltage needed to turn on the relay. When 5V is given to the control circuit, the relay is activated because the high voltage and high current circuit permits current to flow. The relay serves as a connection between the two circuits, ensuring that the control circuit and the high-voltage circuit are kept apart.

#### Water pump

The 12V water pump is a tiny, low-power water pump designed for use in fountains, aquariums, and other aquatic applications. Typically, a 12-V DC power supply powers the pump. Two solar-powered pumps, flow: 4.5 LPM and 3.5 ambers, were employed in the investigation.

#### 12 V battery

12 V battery can generate 12 V of electricity. A battery’s capacity needs to determine how much power it can produce. Ampere-hours (Ah), which indicate the maximum current the battery can provide over a specific period, are frequently used to express capacity. A lead-acid battery (12V, 7.0 AH, Model LP12-7.0, Malaysia) was utilized in this study. When using PV panels in an application, charge controllers are crucial. Disconnecting solar panels when batteries are full avoids overcharging and excessive discharge, maximizing battery efficiency. They also disconnect loads as needed.

#### Solar panel

Solar panels comprise photovoltaic cells, which in experiments have been shown to convert solar energy up to 20% into electrical energy. Nonetheless, most solar panels sold commercially are between 15 and 20% efficient. The specifications of the PV module used in the experiment are detailed in Table 1.

**Table 1:** Specification of solar photovoltaic panel.

Particular	PV module
Maximum power current (Imax)	5.56 A
Maximum power voltage (Vmax)	18 V
Short circuit current (Isc)	5.81 A
Open circuit voltage (Voc)	21.96 V
Maximum power (Pmax)	100 W

#### Voltage sensor

A voltage sensor is a low-complexity device that functions as a voltage divider and is compatible with the Arduino and other microcontrollers running on a 5-V supply. The sensor range used in this research with the microcontroller is 0 to 25 V. Therefore, the following equation can be used to determine the actual output voltage of a photovoltaic panel.

1 $${V}_{out}= {V}_{in}*\frac{{R}_{2}}{{R}_{1}+{R}_{2}}$$

#### Current sensor

Direct and alternating currents can both be measured by the Acs712 current sensor. It is a linear type of sensor. Allegro made this extremely well-known integrated circuit. It offers quick reaction times and noise reduction. There is a 1.5% mistake in the output. With some clever programming, it might be lowered, though, by multiplying the measured value by the standard error of the sensor.

#### Humidity and temperature sensor (DHT11)

Many different control and monitoring applications employ the inexpensive DHT11 temperature and humidity sensor. The DHT11 has a fast response time, excellent stability, and excellent quality. It includes a thermistor to measure temperature, ensuring precise data collection. Its affordability and dependability make it an ideal choice for applications in agriculture, weather monitoring, and other automated systems.

### Measurements

#### PV solar panel performance evaluation

To evaluate the solar panel’s performance during the experimental period, ambient temperature and solar radiation were continuously monitored using precise instruments. A K-type thermometer was used to measure ambient temperature, ensuring accurate readings of the environmental conditions, while solar radiation was measured using a solar meter (model DBTU 1300, USA), providing reliable data on the intensity of solar radiation available throughout the experimental period. This data collection was essential for assessing the panel’s efficiency and performance under varying environmental conditions. The output power (P_output_) was calculated as the product of voltage (V) and current (I), and the PV module’s efficiency was determined as the ratio of electrical output power to solar input power, as described by Eq. (2).

2 $${\eta }_{el}= \frac{{P}_{output}}{{P}_{input}}=\frac{V*I}{A*G}$$

where, A is the PV cell area (m^2^) and G is solar radiation (W/m^2^).

#### Determination of water consumption

This study was conducted in 2023. Daily meteorological data, including minimum and maximum temperatures (Tmin and Tmax, °C), solar radiation (W/m^2^), and wind speed (m/s), were obtained from (https://power.larc.nasa.gov/data-access-viewer/). The daily reference evapotranspiration ($E{T}_{o}$) was calculated using the FAO version 3.2 calculator software^61^, applying the standard FAO Penman method outlined in Allen et al.^62^. The water consumption of the peppermint was determined by utilizing the following equation:

3 $$E{T}_{c. daily}=E{T}_{o}*{K}_{c}$$

The monthly water consumption is calculated using the following equation:

4 $${ET}_{c. monthly}=E{T}_{c. daily}*{m}_{o}$$

Total annual water consumption was obtained by summing the monthly water consumption for all 12 months, as in the following equation:

5 $${ET}_{c. annual}=\sum_{m=1}^{12}{ET}_{c. monthly, m}$$

where, $E{T}_{c}$ is the water consumption of Peppermint (L/m^2^/day), $E{T}_{o}$ is the reference evapotranspiration (mm/day), ${K}_{c}$ is the crop coefficient^63^, ${m}_{o}$ is the number of days in a specific month.

For the smart system, a practical method was employed to estimate the average daily water consumption by observing tank usage over a specific period. This approach assumes that the tank is exclusively used for consumption, with no significant leakage or evaporation. Below are the detailed steps and considerations for this method:

- The experiment began with the tank filled, and the initial volume of water in the tank was noted. This volume was denoted as *V*_*start*_.
- The tank was observed until it was completely emptied. The total water consumed during this period was equivalent to the initial volume (*V*_*start*_).
- The total number of days (*T*) taken to empty the tank completely was recorded.
- The average daily water consumption ($E{T}_{c. daily}$) was calculated using the formula:
  6 $$E{T}_{c. daily}=\frac{{V}_{start}}{T}$$
- Monthly and annual water consumption is measured using Eqs. 4 and 5.

#### Determination of energy consumption of operating pump (*E*_*cp*_)

The daily energy consumption of the pump was calculated using the following formula:

7 $${E}_{cp. daily}=\frac{P * t \boldsymbol{ }\boldsymbol{ }}{\eta }$$

where $P$ is the pump power (W); $\eta$ is the pump efficiency (0.8); and $t$ is the operating time of the pump in hours (h), calculated as:

8 $$t=\frac{E{T}_{c. daily}}{Q}$$

where:

*Q*: The discharge rate of the pump in liters per hour (liter h^-1^). The following equation calculates the monthly energy consumption:

9 $${E}_{cp. monthly}={E}_{cp. daily}*{m}_{o}$$

The annual energy consumption is determined by summing up the monthly energy consumption for all 12 months by the following equation:

10 $${E}_{cp. annual}=\sum_{m=1}^{12}{E}_{cp. monthly, m}$$

#### Determination of peppermint yield

The peppermint yield was estimated individually for each harvest *h*, where *h* = *1,2,3,4*. The total annual yield was then determined by summing the yield of all four harvests.

#### Water-use efficiency (WUE)

WUE is typically evaluated by measuring the weight of the harvested crop and dividing it by the total amount of water used for irrigation. In the context of this research, WUE (kg/L), represents the usable yield of peppermint produced per unit volume of water consumed. This efficiency metric is critical for assessing the effectiveness of water utilization in agriculture. The calculation is performed using the following equation:

11 $$WUE \left(\frac{kg}{L}\right)=\frac{Peppermint \, yield \left(\frac{kg}{{m}^{2}}\right)}{Water \, consumption \left(\frac{L}{{m}^{2}}\right)}$$

#### Energy productivity (EP)

EP is calculated by evaluating the relationship between crop yield and energy consumption, as outlined in the following equation^64^:

12 $$EP \left(\frac{kg}{Wh}\right)=\frac{Peppermint \, yield \left(\frac{kg}{{m}^{2}}\right)}{Energy \, consumption \left(\frac{Wh}{{m}^{2}}\right)}$$

#### Environmental assessment

Greenhouse gases (GHGs) are gases in the atmosphere that trap and release infrared radiation, playing a significant role in the greenhouse effect. Effectively reducing GHG emissions is essential to preserving a safe and sustainable environment. These emissions are a primary factor in climate change, a pressing global issue that has received broad recognition and alarm. In this study, the reduction of CO_2_ emissions resulting from the energy-saving potential of PV modules is calculated using Equation (13). This assessment underscores the vital role of renewable energy technologies in combating climate change and reducing the environmental impact of human activities.

13 $$C{O}_{2} emissions=overall \, consumed \, electrical \, energy*0.45 \left(\frac{kg}{kWh}\right)$$

#### Techno-economic assessment of the smart system

The economic viability assessment holds significant importance as it highlights potential logistical or commercial hurdles a project might encounter, provides remedies for such challenges, and facilitates the gathering and structuring of essential information for project establishment. It is crucial as it demonstrates a project’s capability to attain its set objectives, serving as a guiding tool for investors. By analyzing the outcomes of economic feasibility studies, investors or consumers can pinpoint promising opportunities that warrant further in-depth examination. The economic assessment was conducted under the assumption that the proposed smart irrigation system is designed to support the cultivation of a 211 m^2^ area. All calculations and evaluations were based on this specified cultivation area, ensuring the results accurately reflect the system’s performance and economic feasibility within this operational context.

The life cycle cost (*LCC*) and payback period (*PP*) parameters are used in this study to assess the suggested smart system’s economic feasibility. The payback period is the amount of time needed to recover the initial investment. This formula may be used to determine the smart system’s payback period (*PP*)^65^:

14 $$PP=\frac{TC}{E{C}_{PV}+P}$$

where, $TC$ is the total cost of the smart system, it may be found using the following equation:

15 $${TC=C}_{O}+O\&M$$

The total cost of all the components that make up the smart system, such as the PV panel, storage battery, control unit, charger controller, electric cables, peppermint seedlings, soil, wooden boxes, shipment, and installation, is represented by the term ${C}_{O}$. The expenses for operation and maintenance are *O & M*.

The cost of energy produced by the PV solar system, denoted as $E{C}_{PV}$, is computed using the equation below:

16 $$E{C}_{PV}={E}_{cp. annual}*PE$$

where $${E}_{cp. annual}$$ represents the total energy consumption of the smart system (in kWh), and *PE* is the cost per unit of energy produced by the PV system (in $/kWh). The parameter $${E}_{cp. annual}$$ is determined as:

17 $${E}_{cp. annual}=6 hours*330 sunny days*power of pump \left(kW\right)$$

The number six stands for the number of hours a pump runs daily throughout the year. The suggested smart system’s economic assumptions are displayed in Table 2. The following formula (18) can be used to determine the profits from selling peppermint and the advantages of selling solar energy generated throughout a 25-year life cycle:

**Table 2:** Economic assumptions for the proposed solar PV system to support 211 m^2^ of peppermint cultivation.

Parameters	Assumption
Price of PV panel (100 W)	50 $
Price of battery	20 $
Price of (charger controller + wires)	10 $
Price of (pumps + GR pipes)	30 $
Price of (control unit)	30 $
Price of peppermint seedlings	20 $
Soil (compost + clay + sand)	40 $
Price of wooden boxes	300 $
Price of electricity	0.03 $/kWh
O & M	3% of initial cost
The system life cycle	25 years
Replacement parameters (R)	(Battery, charger controller, wires, pumps, GR pipes, control unit, soil) each 10 years

18 $$Benefits=\left(\left(E{C}_{PV}+P\right)*25\right)-LCC$$

$P$ is the profit from selling an area of 211 m^2^ of peppermint and can be calculated as in the following equation:

19 $$P=yield \left(\frac{kg}{year}\right)*price \left(\frac{\$}{kg}\right)$$

A smart system’s life cycle cost $(LCC)$, may be calculated using the equation that follows^65^:

20 $$LCC= TC+R$$

where *R* represents the total replacement cost, which includes replacing the charger, wiring, pump, control unit, soil, and batteries throughout the system’s lifespan.

## Results and discussions

### PV module performance analysis

**Fig. 8:**
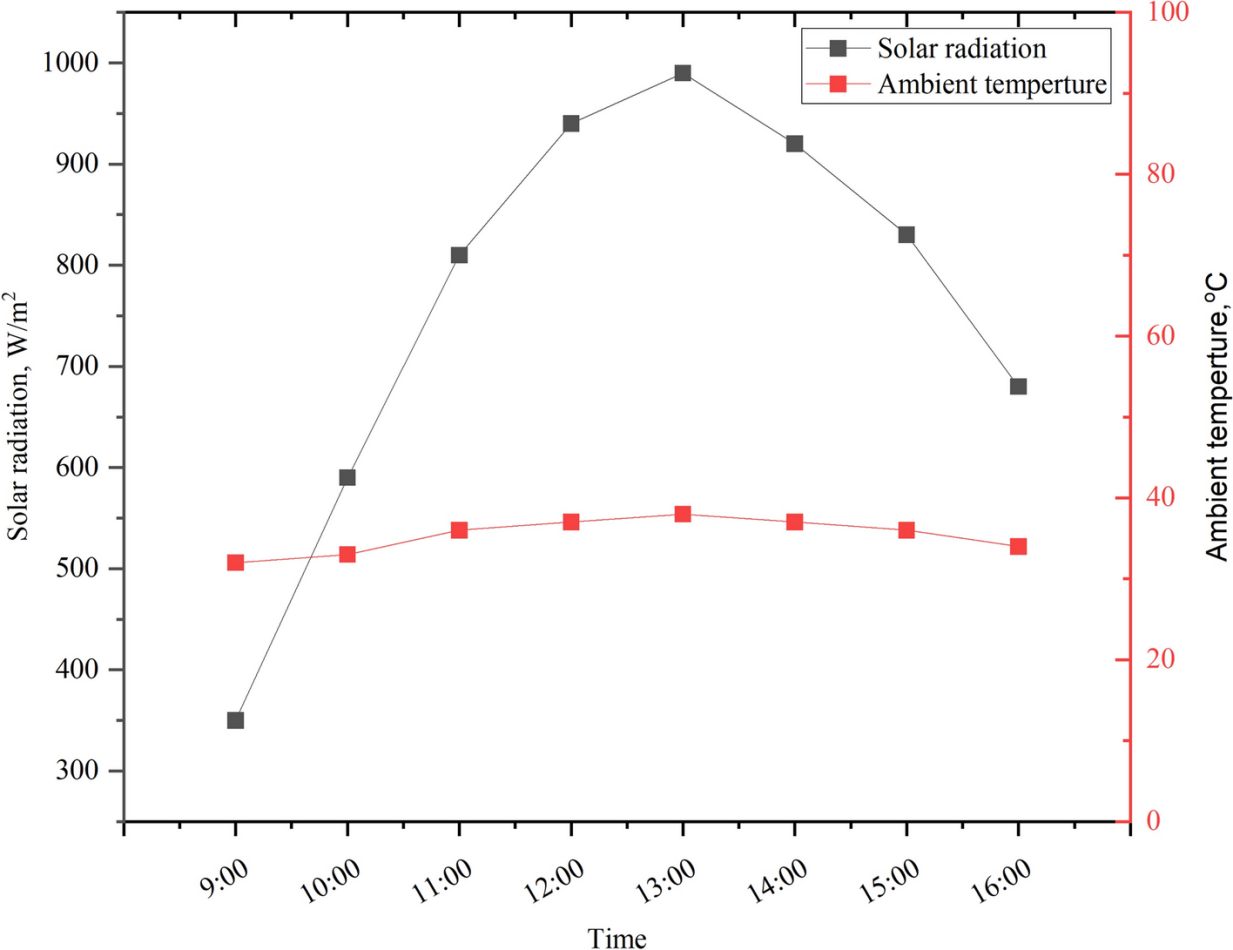
The solar radiation and the ambient temperature of an experimental day in June 2023.

This study adopted the drip irrigation system, which may be utilized for both building rooftop gardens and agricultural purposes. Figure 8 shows the solar radiation and ambient temperature of an experimental day in June 2023. For the study region, the PV system’s performance analysis is examined. The PV module-generated voltage and current are fed into the Arduino controller. Sensors attached to the Arduino controller are used to measure the temperature of the module and the ambient temperature. Upon connecting the Arduino board to the computer via a USB cable, the Excel Spreadsheet will gather and present the output data in real time, as seen in Table 3.

**Table 3:** A real-time virtual instrumentation system paired with a sample of experimentally acquired data.

Time	Moisture content (%)	Temperature (°C)	RH (%)	Voltage (V)	Current (Amper)	Power (W)
12:38:02.00	70	48.4	21.4	21.5	4.6	98.9
12:38:03.00	70	48.5	21.2	21.5	4.6	98.9
12:38:04.00	70	48.5	21.2	21.5	4.6	98.9
12:38:05.00	70	48.6	21.1	21.5	4.6	98.9
12:38:06.00	70	48.7	21.1	21.5	4.6	98.9
12:38:07.00	70	48.7	21	21.5	4.6	98.9
12:38:08.00	71	48.8	21	21.5	4.6	98.9
12:38:09.00	71	48.8	20.9	21.5	4.6	98.9
12:38:10.00	71	48.9	20.8	21.5	4.6	98.9
12:38:11.00	74	49.0	20.9	21.5	4.6	98.9
12:38:12.00	74	49.0	20.8	21.5	4.6	98.9
12:38:13.00	74	49.1	20.7	21.5	4.7	101.0
12:38:14.00	75	49.1	20.6	21.6	4.7	101.5
12:38:15.00	75	49.2	20.6	21.6	4.7	101.5
12:38:16.00	76	49.3	20.5	21.6	4.7	101.5

**Fig. 9:**
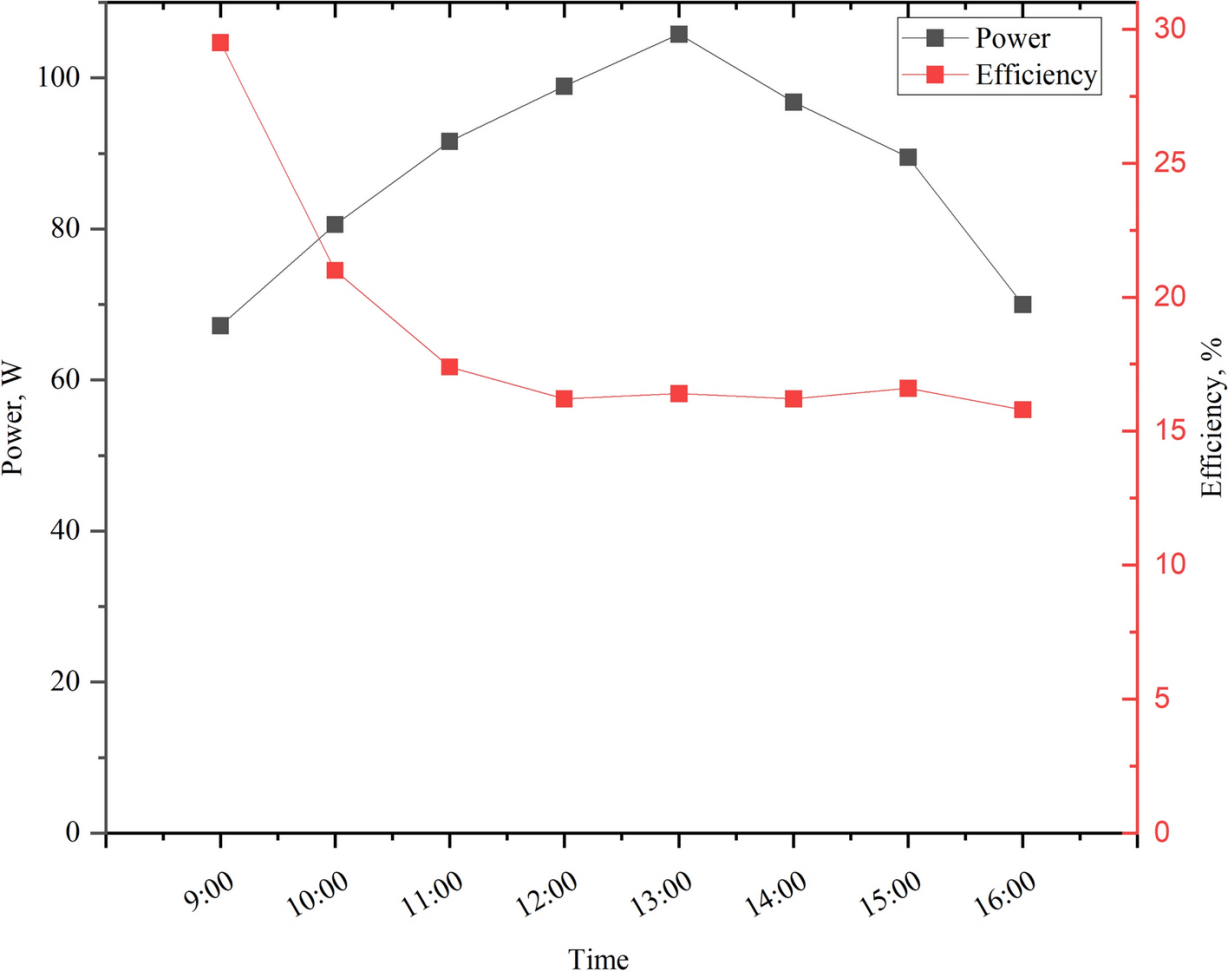
Average hourly variation of power and electrical efficiency during a typical day in June 2023.

Figure 9 illustrates the variations in the output power of the PV panel under investigation throughout the day. The data reveals that the maximum output power of 105 W is achieved at 13:00, coinciding with peak ambient temperature and solar radiation levels. Electrical efficiency is a critical parameter for assessing PV system performance; however, reporting transient variations in efficiency throughout the day is less conclusive due to simultaneous fluctuations in solar irradiation and output power. These dynamic changes affect both the numerator and denominator of the efficiency calculation, making instantaneous values less reliable for evaluation. Instead, the daily average values provide a more comprehensive measure, with the PV panel achieving an average output power of 87.5 W and an electrical efficiency of 18.6%. This approach highlights the system’s overall performance under varying environmental conditions.

#### Water, energy, and yield analysis

**Fig. 10:**
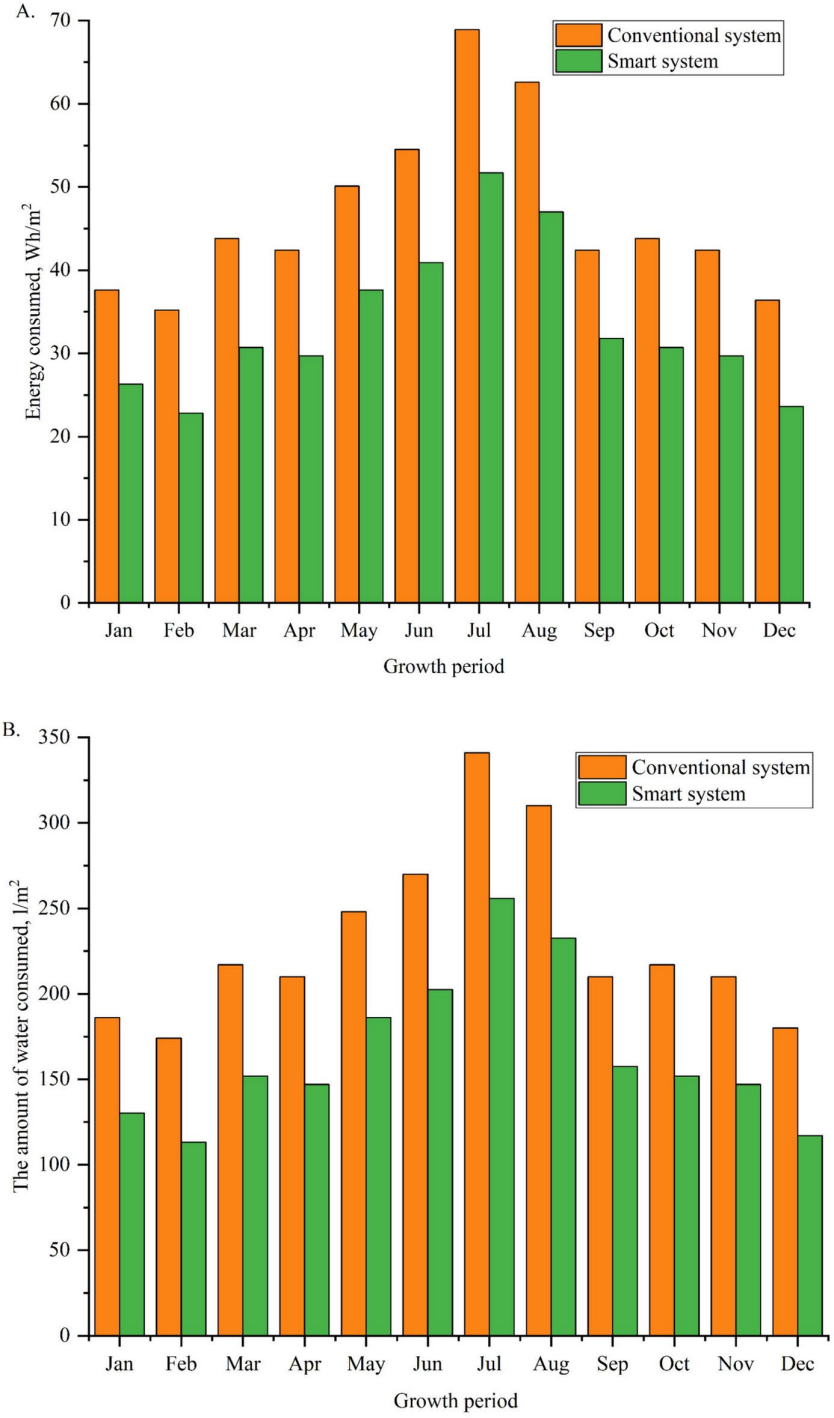
Monthly variation of (**A**) energy consumption, and (**B**) irrigation water consumption for conventional and smart systems.

The energy and water consumption of both the conventional and the smart irrigation systems were monitored and analyzed to compare their performance. The monthly energy consumption of the two systems is plotted in Fig. 10A. The maximum energy consumed in the conventional system and the smart system obtained during July was 68.9 and 51.7 Wh/m^2^. Conversely, February witnessed the minimum energy consumption, with values of 35.2 Wh/m^2^ for the conventional system and 22.8 Wh/m^2^ for the smart system. This reduction in energy usage demonstrates the efficiency of the smart system, attributed to its optimized operations based on real-time soil moisture monitoring.

Similarly, the monthly water consumption is illustrated in Fig. 10B. The conventional irrigation system consumed a maximum of 341 L/m^2^ in July, while the smart system consumed 255.7 L/m^2^ during the same period. In February, water consumption reached its lowest, with 174 L/m^2^ for conventional irrigation and 113.1 L/m^2^ for the smart system. These results highlight the significant water-saving potential of the smart system, which adapts irrigation schedules to actual soil moisture levels. The findings align with similar studies emphasizing the benefits of smart irrigation systems. For instance, Sharifnasab et al.^66^ demonstrated that smart irrigation systems integrated with soil moisture sensors could reduce water consumption by up to 35% compared to conventional methods, closely matching the 28.1% reduction observed in this study. Likewise, Garcia et al.^67^ reported energy savings of between 20 and 29% in solar-powered smart irrigation systems, corroborating the 28.1% reduction in energy use found here. Furthermore, these results reinforce the importance of real-time monitoring and automation in reducing resource usage. The reduced energy and water consumption observed in the smart system improves sustainability and aligns with global efforts to conserve natural resources and mitigate climate change impacts. These comparisons underline smart irrigation technologies’ growing relevance and effectiveness in modern agriculture.

**Fig. 11:**
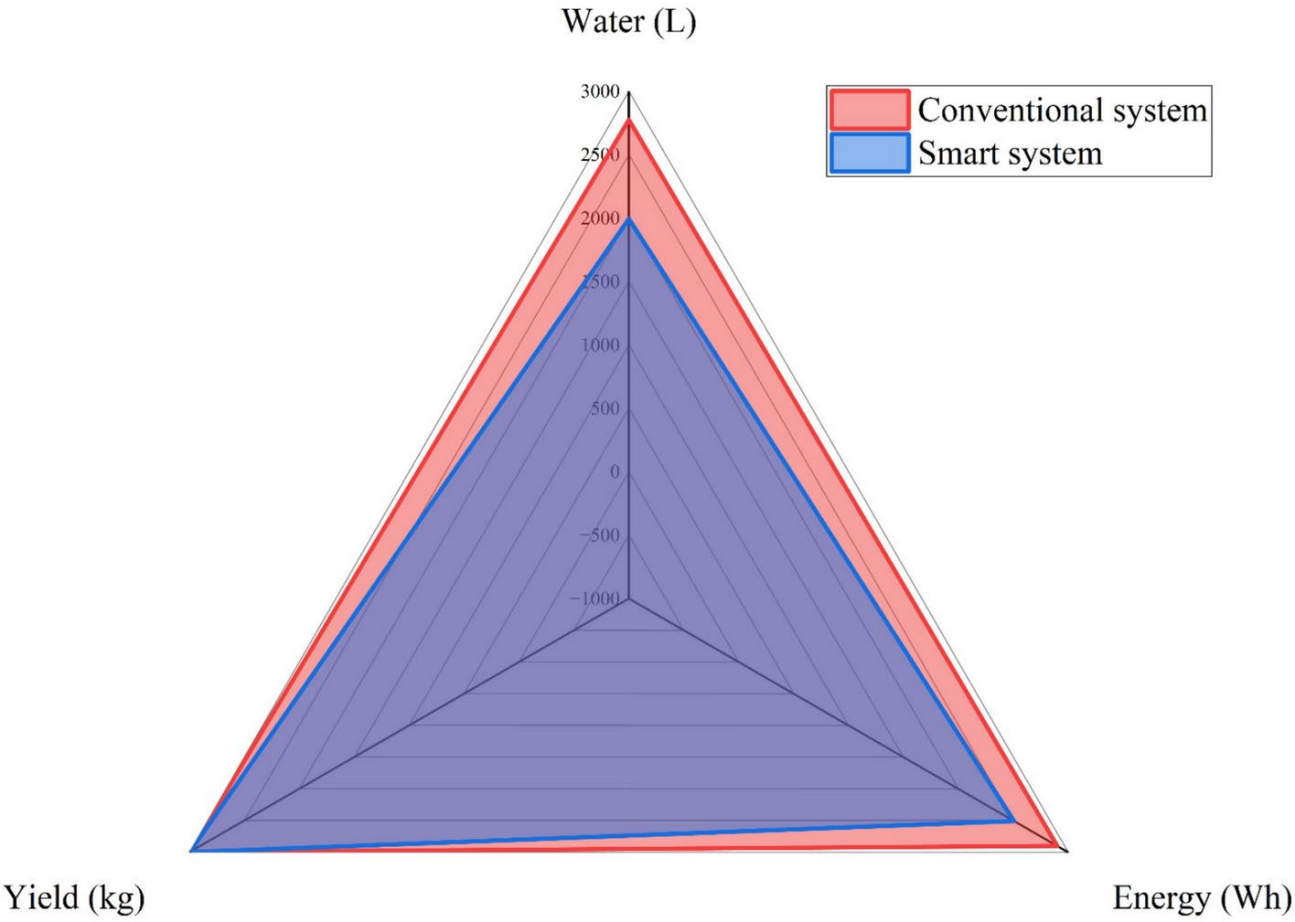
Annual variation of energy consumption, irrigation water consumption, and yield for conventional and smart systems.

The reduction in water and energy consumption achieved by the smart irrigation system compared to conventional system was measured through a mix of testing and observation. Figure 11 highlights the reduced usage of these critical resources by the smart system. A detailed comparison was conducted, analyzing the data collected from both irrigation systems over the entire growing season. The reduction in water or energy consumption can be calculated by deducting the amount of water or energy used in a smart irrigation system from the amount used in a conventional system and dividing the result by the amount used in a conventional system. For water saving, the smart irrigation system consumed 1992.3 L/m^2^/year compared to 2773 L/m^2^/year for conventional system. (2773–1992.3)/2773 is the outcome of 0.281 or 28.1% of the total. The calculation of energy savings involves deducting the energy consumption of the smart irrigation system (402.5 Wh/m^2^/year) from the energy consumption of the conventional system (560.2 Wh/m^2^/year). (560.2–402.5)/560.2 is the outcome, or 0.281, or 28.1% of the total. There are several obstacles to reducing irrigation water usage by more than 28.1%, including possible effects on crops, environmental factors, adapting to climate change, managing soil moisture effectively, technology constraints, and farmer education. The smart system can save more water and energy than was used to be applied on a larger scale, so supplying solar energy for irrigation is a viable solution. The harvested peppermint quantity remained identical for both the conventional and smart irrigation systems. This observation is a critical point of the study, emphasizing that while irrigation methods significantly impact resource efficiency, they did not adversely affect the harvest yield. This finding underscores the potential of smart irrigation systems to optimize water and energy usage without compromising crop productivity, thereby maintaining agricultural output while promoting sustainability.

#### WUE, EP, CO_2_ emissions, and economic analysis

**Fig. 12:**
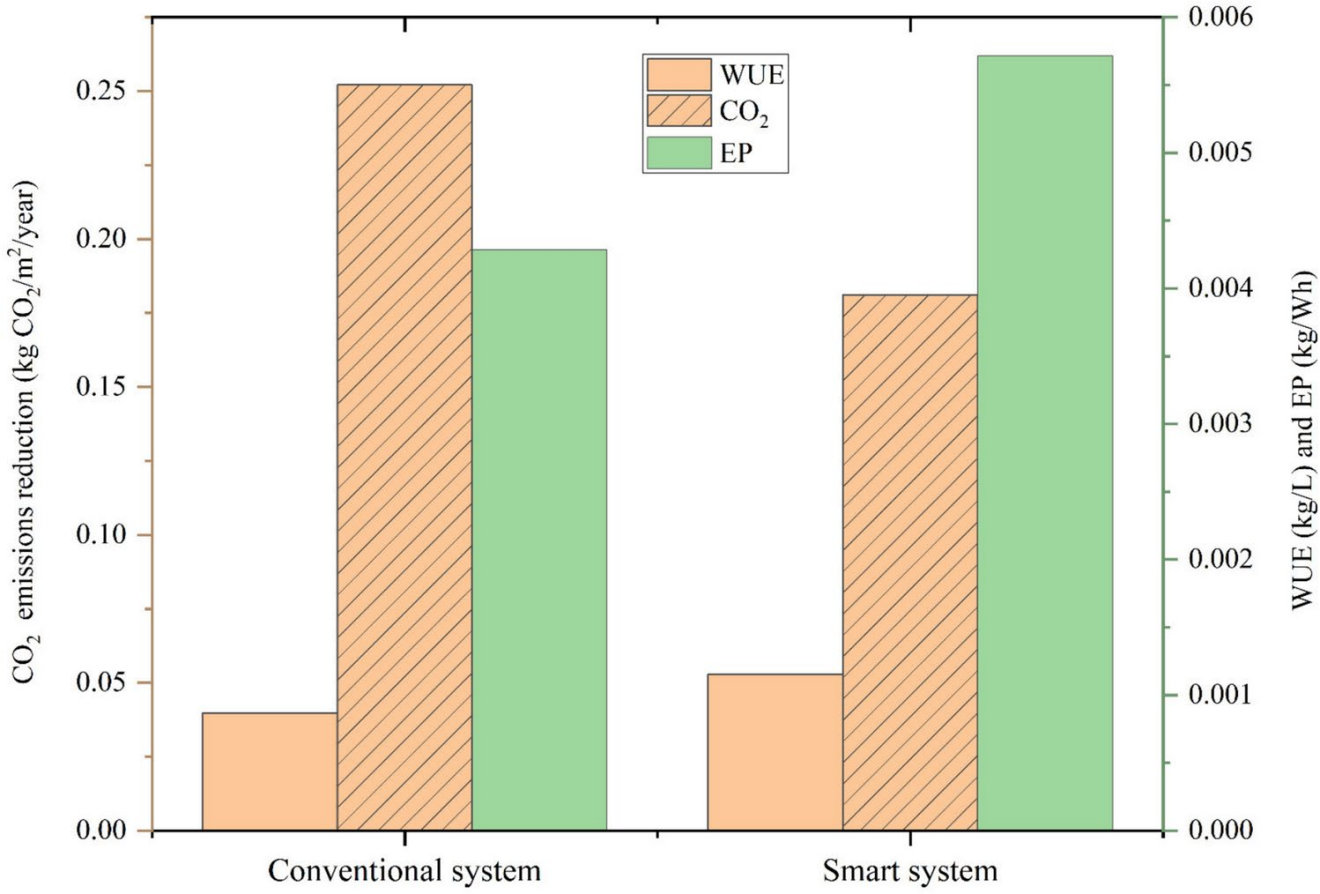
WUE, EP, and CO_2_ emission reduction for the conventional and smart systems.

The WUE and EP values for both the conventional and smart systems are plotted in Fig. 12. The smart system led to an increase in WUE and EP, whereas conventional irrigation showed the lowest WUE and EP values. The highest WUE and EP values were observed in plants grown under the smart system, and the values were 0.0011 kg/L and 0.0057 kg/Wh for WUE and EP, respectively, representing superior performance in resource utilization. Conversely, the lowest WUE and EP values were observed in plants grown under conventional irrigation, and the values were 0.00087 kg/L and 0.00428 kg/Wh for WUE and EP, respectively. These results highlight the effectiveness of the smart system in optimizing water and energy usage for agricultural production.

The annual CO_2_ emission reduction values were calculated for the conventional irrigation and smart system and plotted in Fig. 10. Conventional irrigation and smart systems have led to decreased CO_2_ emissions. The highest CO_2_ emission reduction was observed under the smart system, and the CO_2_ emission reduction values were 0.252 and 0.181 kg CO_2_/m^2^/year for the conventional irrigation and smart system, respectively. In addition, the smart system reduced the carbon footprint by 28.1%, higher than conventional irrigation. The integration of solar energy into irrigation systems offers significant advantages, extending beyond the elimination of electricity costs—a growing concern that challenges the economic viability of irrigation for many farmers^68^. It also contributes to substantial environmental benefits by reducing CO2 emissions^69^. These results underscore the potential of smart irrigation systems to address both resource scarcity and environmental concerns. The smart system’s superior performance can be attributed to its real-time monitoring and control capabilities, which ensure precise water and energy application. Additionally, its use of photovoltaic energy reduces reliance on non-renewable resources, further contributing to sustainability.

The economic study was conducted by observing the pricing in Egypt over the past several years. This assessment incorporated the real investment cost of all system components, including estimated replacement costs, to provide a comprehensive understanding of the system’s economic sustainability. Table 4 displays the installation and operational expenses for the smart system across its 25-year lifecycle. Although the initial installation cost was relatively high, the total life cycle cost was calculated to be 775$. Furthermore, the economic payback period was estimated to be 5.6 years, demonstrating a reasonable return on investment. This range is often influenced by local energy prices, installation costs, and the scale of the system. The 775$ lifecycle cost of the system, when amortized over 25 years, represents a cost-effective solution for water and energy management, particularly in regions facing resource scarcity. A solar-powered smart irrigation system offers substantial long-term cost savings and economic advantages while contributing to broader environmental and societal benefits. By eliminating recurring electricity costs and optimizing water usage through smart sensors, the system significantly reduces operational expenses. Automation further cuts labor costs, while efficient water management extends equipment lifespan and minimizes maintenance expenses. Additionally, by decreasing reliance on grid electricity and fuel, the system provides resilience against energy price volatility, ensuring more predictable operating costs for urban farmers. Increased agricultural productivity from precise irrigation leads to higher yields and improved profitability. Beyond direct financial savings, the system enhances urban food security by supporting local food production, reducing transportation costs, and lowering dependency on imported produce. Farmers may also benefit from carbon credit programs and government incentives for adopting renewable energy solutions. Over time, the system’s cost savings, combined with sustainability advantages, make it a financially viable and environmentally responsible investment, promoting resource efficiency and long-term economic resilience.

**Table 4:** Economic analysis of the smart system (in $) for a 25-year lifespan to produce a 211 m^2^ of peppermint.

Parameter	Cost ($)
Initial cost	500 $
O & M	15 $
R	130 $
LCC	775 $
EC_PV_	2.4 $/year
Profit	101.2 $/year
Benefit	2597.7 $/25 years

Table 5 shows different studies for a comparative analysis based on smart solar irrigation, highlighting the main functions and crops used and their ability to reduce water and energy consumption. The comparison articles were chosen for inclusion based on their applicability for tracking energy and water consumption. Studies^39–43^ focused on monitoring soil moisture using sensors. In comparison, our paper differs by using solar energy to operate pumps with smart irrigation, and our study has already been applied to peppermint, taking results and readings, calculating the amount of water and energy consumed, and coming up with recommendations, presenting a more advanced and comprehensive approach to water and energy conservation.

**Table 5:** Comparison of related studies.

References	Smart irrigation	Solar energy	Crop/simulation	Water reduced	Energy reduced
Wang et al.^42^	Yes	No	Simulation	No	No
Morchid et al.^41^	Yes	No	Simulation	70%	No
Blessy and Kumar^39^	Yes	No	Simulation	No	No
Hamouda et al.^40^	Yes	No	Trees	50%	No
Ismail et al.^54^	Yes	Yes	Simulation	No	No
Dey et al.^55^	Yea	Yes	Rice	No	No
Ravikumar et al.^56^	Yes	Yes	Simulation	No	No
Sinugo and Longe^58^	Yes	Yes	–	25%	57.8%
Wanyama et al.^51^	Yes	Yes	Simulation	No	No
Yadav et al.^59^	Yes	Yes	Potato	9.24%	9.24%
Proposed system	Yes	Yes	Peppermint	28.1%	28.1%

The studies^51,54,56^ used smart irrigation with solar energy. Our study has already been applied to peppermint, calculating the amount of water and energy consumed by 28.1%. This research successfully transforms conventional methodologies by articulating a breakthrough in irrigation accuracy and efficiency. It presents a solar-powered smart irrigation system that uses 28.1% less water than the industry standard, making it environmentally beneficial. The study examines broader implications for global food security and agricultural sustainability, emphasizing the noteworthy improvements in precision and efficiency made possible by the recommended smart irrigation system. Since access to water reduces poverty, using water resources wisely and fairly through scientifically supported WEF-nexus irrigation technology increases water reliability and sufficiency at all scales. In addition to supporting several sustainable development goals, the WEF nexus strategy may be used to ensure food, energy, and water security in resource-poor areas.

### Strategies for improving sustainable resource management within the solar-agriculture-water (SAW) nexus’s

Rigorous regulations have demonstrated a favorable impact on carbon emissions and sustainability^70^. These policies require meticulous examination and execution to pave the way for a sustainable future. The strategy should be all-encompassing, considering the interconnectedness and trade-offs between sectors, emphasizing optimizing resource utilization, waste reduction, and resilience enhancement. The following policy recommendations are put forward to advance sustainable resource management:

1. Advocating for integrated planning strategies that acknowledge the interrelations among water, energy, and food systems, and foster stakeholder cooperation.
2. Fostering knowledge and understanding of the benefits of sustainable resource management in the solar, agricultural, and water domains among stakeholders, such as farmers, legislators, and the general public.
3. Creating legislative frameworks that support sustainable resource management techniques, including water use licenses or building energy efficiency requirements.
4. Creating land lease contracts with solar developers that give non-agricultural areas such as barren or degraded forests priority for solar projects to relieve pressure on agricultural areas.
5. Enforcing water price regulations that account for the true cost of water use, including delivery, treatment, and environmental effects. This might encourage sustainable water management techniques and encourage solar producers to utilize water more wisely^71^.
6. Promoting and rewarding the use of robotic cleaning systems for solar panels as a way to save labor expenses and replace water use.

## Conclusions

This study introduces an innovative integration of solar-powered smart irrigation systems for sustainable urban agriculture, emphasizing water conservation, energy efficiency, and a reduction in carbon emissions. This research demonstrates a significant advancement over traditional irrigation methods by designing and implementing a rooftop irrigation system equipped with real-time monitoring and control capabilities. The results indicate that the proposed system achieved a 28.1% reduction in water consumption, alongside a notable decrease in CO₂ emissions of 0.252 kg CO₂/m^2^/year, underscoring its potential for climate adaptation and environmental sustainability. Beyond immediate water and energy savings, this system contributes to the broader water-energy-food nexus, addressing critical challenges in densely populated urban areas where space and resources are limited. The findings provide a promising pathway for resource-efficient food production in urban settings, supporting the shift toward resilient, climate-smart agricultural practices.

Furthermore, the economic analysis indicates that the system is both environmentally beneficial and economically viable, with a reasonable payback period, making it an attractive option for urban agriculture stakeholders. This research adds valuable insights into the feasibility of rooftop agriculture supported by renewable energy and highlights the potential for scaling such systems in diverse urban environments. The deployment of this technology could revolutionize urban agriculture by providing a scalable, eco-friendly solution that promotes food security and reduces reliance on traditional energy sources. Future research should focus on enhancing system efficiency through advanced sensors and weather analytics, integrating additional renewable energy sources, and testing scalability across diverse urban environments. Life cycle assessments and machine learning for predictive maintenance could further optimize performance, solidifying solar-powered smart irrigation as a sustainable urban agriculture solution.

## Data Availability

Data available on request from corresponding author mahmoudabdelhamid@agr.asu.edu.eg.
